# Reproductive dysfunction in hemodialysis: endocrine mechanisms, clinical features, and therapeutic approaches

**DOI:** 10.1080/0886022X.2025.2565406

**Published:** 2025-10-09

**Authors:** Wei Gou, Cheng Xue, Fanzhou Zeng, Changhao Zhu, Bo Yang

**Affiliations:** ^a^ Department of Nephrology, Naval Medical Center of PLA, Naval Medical University, Shanghai, China; ^b^ Division of Nephrology, Changzheng Hospital, Naval Medical University, Shanghai, China

**Keywords:** Reproductive dysfunction, hemodialysis, hypothalamic-pituitary-gonadal axis, sexual dysfunction, hypogonadism, infertility

## Abstract

Reproductive dysfunction is a near-universal and debilitating complication in patients on maintenance hemodialysis, profoundly impacting quality of life. The core patho­physiology is a ‘dual-hit’ endocrine collapse, where the uremic milieu causes both central suppression of the hypothalamic-pituitary-gonadal (HPG) axis and peripheral gonadal resistance. In men, this manifests as hypogonadism, with erectile dysfunction serving as a critical sentinel marker for systemic vascular disease. In women, it results in a reversible menopause-like state characterized by anovulation and infertility. While conventional hemodialysis is insufficient to correct these derangements, practical therapeutic strategies can mitigate them. Intensified regimens like nocturnal hemo­dialysis can partially restore fertility and dramatically improve pregnancy outcomes. Targeted pharmacological interventions, including phosphodiesterase-5 inhibitors and hormone replacement therapy, are also effective for specific indications. Kidney transplantation offers the best chance of restoring normal endocrine function. Future directions include novel therapeutics targeting the kisspeptin system and the development of wearable artificial kidneys to provide continuous uremic clearance. This review provides a clinically focused overview of the endocrine mechanisms, features, and management of reproductive dysfunction in hemodialysis.

## Introduction: the overlooked burden of reproductive dysfunction in hemodialysis

### Defining the clinical problem

Reproductive and sexual dysfunction represent a profound and often underappreciated complication of end-stage renal disease (ESRD) and its management with hemodialysis (HD). Far from being a peripheral concern, this dysfunction is a central component of the uremic syndrome, deeply intertwined with the patient’s quality of life, psychological well-being, and interpersonal relationships [1,2]. The clinical presentation is broad, encompassing diminished libido, erectile dysfunction (ED), impaired spermatogenesis, and infertility in men, alongside anovulation, menstrual irregularities, and infertility in women [3,4]. The etiology is decidedly multifactorial, representing a complex interplay of the physiological derangements of uremia, the psychological burden of chronic illness, associated comorbidities such as vascular disease, and the iatrogenic effects of medications [5,6]. While each of these components plays a role, the core pathogenic driver is the profound and systemic disruption of the endocrine system, particularly the hypothalamic-pituitary-gonadal (HPG) axis, by the uremic state [7]. Addressing this dysfunction is therefore not an ancillary aspect of care but is crucial for the holistic management of the patient on hemodialysis.

### The epidemiological landscape: a pervasive and under-recognized complication

The prevalence of reproductive dysfunction in the hemodialysis population is remarkably high, establishing it as a near-universal complication of ESRD rather than an infrequent occurrence. The scale of the problem underscores its clinical significance and the urgent need for greater recognition and management.

In male patients, sexual dysfunction is exceptionally common. The prevalence of ED is consistently reported in the range of 76% to 87% among men undergoing maintenance hemodialysis, a figure that dwarfs rates in the general population [8]. A comprehensive meta-analysis further solidified this finding, reporting that approximately 70% of men with chronic kidney disease (CKD) experience some form of self-reported sexual dysfunction [9]. The severity of this dysfunction appears to correlate with the decline in renal function, worsening as patients progress through the stages of CKD toward dialysis dependence.

Among female patients, the burden of sexual dysfunction is similarly severe, though historically less well-researched [10]. Meta-analytic data and cohort studies indicate that sexual dysfunction affects a staggering 80% of women on hemodialysis [11]. The clinical manifestations are varied and distressing, commonly including diminished libido, difficulties with arousal and orgasm, vaginal dryness, and pain during intercourse (dyspareunia) [12,13].

A critical observation emerges when comparing the prevalence of sexual dysfunction across different modalities of renal replacement therapy (RRT). A systematic review and meta-analysis provided compelling evidence of a gradient of dysfunction. The prevalence of sexual dysfunction was highest in patients on hemodialysis (80%), followed by those on peritoneal dialysis (PD) at 67%, and was lowest in renal transplant recipients at 63% [11]. This pattern is not merely a statistical curiosity; it strongly suggests a causal, dose-dependent relationship between the intensity of the uremic state and the severity of reproductive impairment. Kidney transplantation, which offers the most complete restoration of renal metabolic and endocrine clearance, is associated with the best reproductive outcomes [14]. Conversely, conventional intermittent hemodialysis, which allows for significant interdialytic accumulation of uremic toxins, is associated with the most severe dysfunction. This relationship underscores that the retained solutes and the inflammatory milieu of uremia are likely the primary, and at least partially reversible, drivers of the endocrine collapse that underpins reproductive failure in this population. The efficacy of an RRT modality in mitigating this uremic state appears to be a direct predictor of the patient’s potential for reproductive health.

### The centrality of endocrinology

This review will posit that the constellation of reproductive failures observed in hemodialysis patients stems fundamentally from a profound derangement of the body’s endocrine control systems. The uremic state wages a multi-pronged assault on the HPG axis, disrupting its intricate feedback loops and pulsatile signaling. This leads to a cascade of hormonal abnormalities that directly impair gonadal function, resulting in the clinical syndromes of hypogonadism in men and anovulatory infertility in women [15–17]. Understanding this endocrine collapse is paramount to developing rational and effective therapeutic strategies to restore not just a single function, but a critical aspect of human health and well-being for this vulnerable patient population.

BOX: methods

- This narrative review synthesizes evidence on the endocrinology of reproductive dysfunction in hemodialysis.
- A literature search was conducted using PubMed and Google Scholar for articles published up to August 2025. Search terms included ‘hemodialysis’, ‘end-stage renal disease’, ‘reproductive dys­function’, ‘sexual dysfunction’, ‘hypogonadism’, ‘infertility’, ‘erectile dysfunction’, ‘amenorrhea’, ‘hypothalamic-pituitary-gonadal axis’, ‘prolactin’, and ‘kisspeptin’.
- Inclusion criteria focused on clinical trials, meta-analyses, systematic reviews, and key observational studies relevant to the pathophysiology, clinical presentation, and management of these conditions.
- Evidence was prioritized based on study design, with meta-analyses and randomized controlled trials given the highest weight.

## The pathophysiological core: uremia and the disintegration of the hypothalamic-pituitary-gonadal axis

The reproductive dysfunction seen in hemodialysis patients is not a consequence of a single organ failure but rather the systemic manifestation of the uremic syndrome. The failure of the kidneys to excrete metabolic waste products, maintain electrolyte and acid-base balance, and perform their endocrine functions creates a hostile internal milieu that disrupts physiological processes throughout the body, with the HPG axis being particularly vulnerable (Table 1).

**Table 1.** Neuroendocrine hormonal disruptions in hemodialysis.

Hormones	Specific alterations	Mechanism	Clinical consequence
Hypothalamus-Pituitary (GnRH, LH, FSH)	Loss of pulsatile GnRH signaling; ↑ LH half-life; ↑ baseline LH & FSH, blunted FSH/LH ratio	Reduced renal clearance, Uremic toxins (e.g., β2-microglobulin, cytokines) disrupt neurotransmission; impaired feedback regulation	Hypergonadotropic hypogonadism: testicular failure, ovarian anovulation, luteal defects
Prolactin	↑ Serum prolactin levels (30–65% prevalence)	Reduced renal clearance + decreased dopaminergic inhibition	Inhibition of GnRH → amenorrhea, low libido, erectile dysfunction, galactorrhea
Sex hormones	Men: ↓ total/free testosterone; Women: absent cyclical estradiol and progesterone rises	Direct uremic and inflammatory effects on gonads; receptor resistance	Spermatogenic failure (oligospermia/azoospermia), amenorrhea, anovulation, vaginal atrophy
Adrenal & binding proteins	↓ Dehydroepiandrosterone sulfate; stable sex hormone binding globulin levels	Inflammation; malnutrition; failure of adrenal androgen production	A low DHEA-S level was a predictor of all-cause mortality in male

DHEA-S: dehydroepiandrosterone-sulfate; FSH: Follicle-Stimulating Hormone; GnRH: Gonadotropin-Releasing Hormone; LH: Luteinizing Hormone.

### The uremic milieu: a multi-pronged assault on endocrine function

The uremic state is defined by the retention of a vast array of solutes that are normally cleared by healthy kidneys. These ‘uremic toxins’ are the primary effectors of the syndrome’s pathophysiology [18,19]. They are broadly classified into small water-soluble compounds (e.g., urea), protein-bound solutes (e.g., indoxyl sulfate), and middle molecules [19,20]. These toxins exert deleterious effects by disrupting fundamental cellular processes, altering gene expression, and interfering with critical enzymatic and hormonal signaling pathways, including those that govern the HPG axis [18,21–23]. Certain substances that accumulate in uremia, such as prolactin, are considered to be functional uremic toxins themselves due to their direct inhibitory effects on reproductive hormone secretion [24].

Compounding the direct toxicity of retained solutes, uremia is intrinsically a state of chronic, low-grade inflammation and heightened oxidative stress, sometimes referred to as the malnutrition-inflammation-atherosclerosis (MIA) syndrome [25,26]. The accumulation of uremic toxins and factors related to the dialysis procedure itself (e.g., bio-incompatible membranes) stimulate the production of pro-inflammatory cytokines like tumor necrosis factor-alpha (TNF − α) and interleukin-6 [27,28]. This inflammatory cascade, along with an imbalance in reactive oxygen species (ROS) production and antioxidant defenses, promotes systemic endothelial dysfunction—a key pathogenic factor in vasculogenic ED—and can directly impair steroidogenesis in the gonads and disrupt hormone secretion from the pituitary gland [29].

### Central dysregulation: chaos at the command center

The intricate, rhythmic communication within the HPG axis begins in the brain, and it is here that uremia delivers a critical blow. The foundational defect in uremic reproductive dysfunction is the disruption of the normal, pulsatile secretion of gonadotropin-releasing hormone (GnRH) from the hypothalamus. This rhythmic pulse is essential for stimulating the pituitary gland to release gonadotropins in the correct pattern. In uremia, this pulse generator is suppressed and disorganized, likely due to the neurotoxic effects of retained solutes and the modulatory influence of inflammatory cytokines [30–32].

The pituitary gland, in turn, exhibits an aberrant response. In men, while baseline levels of luteinizing hormone (LH) are often high, the amplitude of the secretory pulses is blunted, resulting in a less potent biological signal to the testes. In women, the consequences are even more dramatic: the crucial mid-cycle surge of LH, which is the absolute requirement for triggering ovulation, is completely abolished. The fact that the pituitary gland responds abnormally even when stimulated directly with exogenous GnRH indicates that the dysfunction is not solely due to a lack of hypothalamic input but also involves an intrinsic pituitary defect [32–34].

### Peripheral failure: resistance and damage at the gonads

The disruptive signals from the brain are met with impaired responses and direct damage at the level of the gonads. In men, the uremic state induces a state of primary testicular failure. The Leydig cells, which produce testosterone, become resistant to the circulating LH, resulting in impaired testosterone synthesis despite high LH levels. Simultaneously, the Sertoli cells and germinal epithelium within the seminiferous tubules are damaged, leading to impaired sperm production (spermatogenesis) [16]. Testicular biopsies from uremic men frequently show evidence of this damage, including germ-cell aplasia, arrested sperm development (hypospermatogenesis), and tissue scarring (fibrosis) [35].

In women, the situation is more nuanced. While the ovary itself seems to retain its intrinsic functional capacity—it can be stimulated to produce estrogen with medications like clomiphene—the chronically disordered hormonal environment renders it nonfunctional. The absence of the cyclical LH surge means that ovarian follicles fail to mature and rupture, a state of persistent anovulation. This effectively creates a ‘reversible menopause-like state’, where the ovaries are capable but are never given the correct signals to perform their cyclical duties [36].

**Figure 1.**
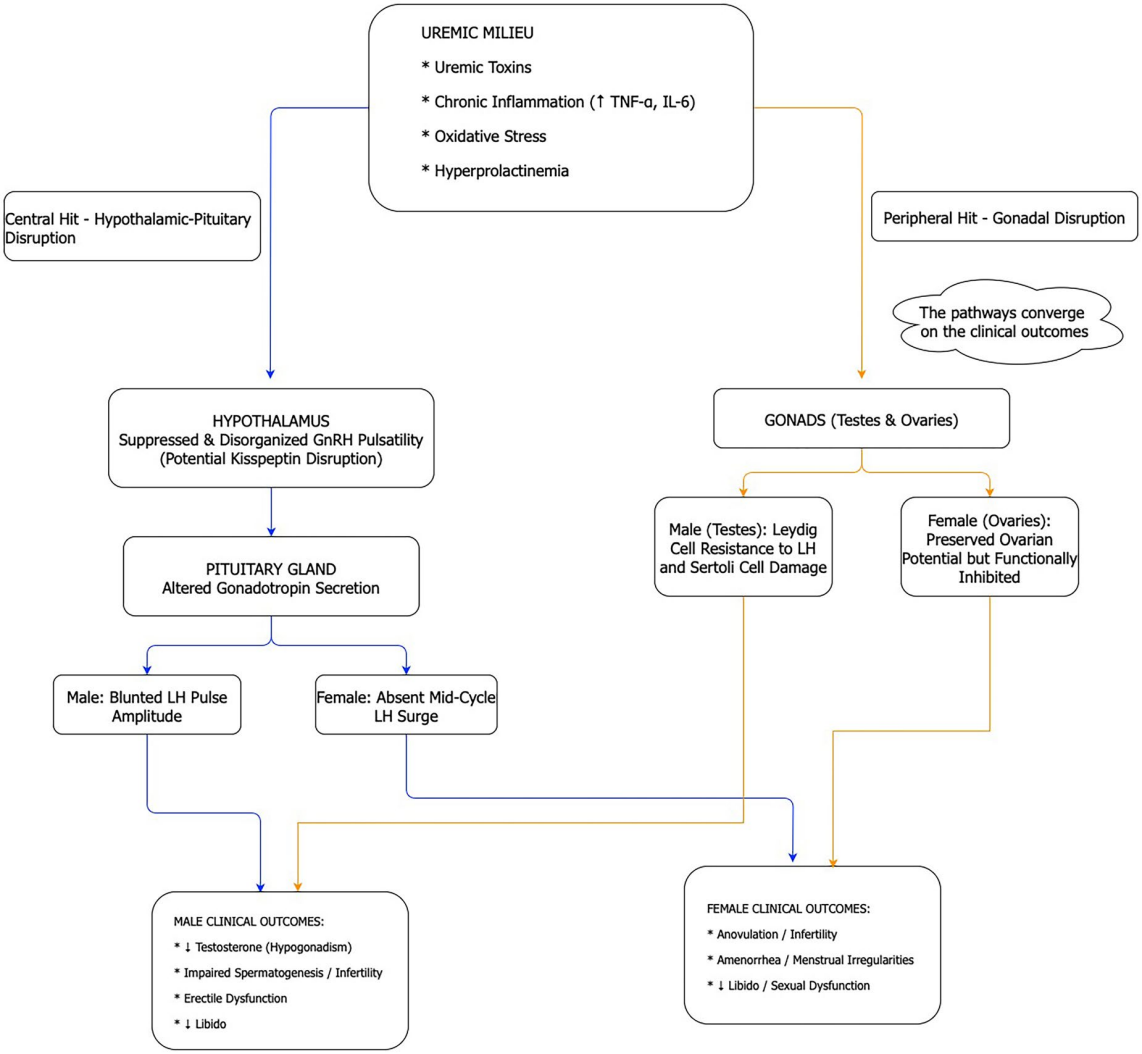
Pathophysiology of the ‘dual-hit’ disruption of the hypothalamic-pituitary-gonadal (HPG) axis in uremia. The uremic state induces reproductive dysfunction through two primary mechanisms [1]. Central Disruption: Uremic toxins, chronic inflammation, and hyperprolactinemia suppress the hypothalamic GnRH pulse generator. This leads to aberrant pituitary signaling, characterized by blunted LH pulsatility in men and a complete absence of the mid-cycle LH surge in women [2]. Peripheral Disruption: Uremic toxins and oxidative stress also exert direct negative effects on the gonads, impairing steroidogenesis and gametogenesis. This dual disruption results in distinct clinical syndromes: hypergonadotropic hypogonadism in men and a state of anovulatory infertility, or menstrual irregularities in women. Abbreviations: GnRH, gonadotropin-releasing hormone; LH, luteinizing hormone; FSH, follicle-stimulating hormone.

The pathophysiology of uremic hypogonadism is therefore not a simple, linear pathway of failure but a more complex ‘dual-hit’ phenomenon. Uremia simultaneously cripples the HPG axis at both the central command level (hypothalamus and pituitary) and the peripheral effector level (gonads). A purely central defect would typically present with low gonadotropin levels (LH and FSH, luteinizing hormone and follicle-stimulating hormone), while a purely primary gonadal failure would result in markedly elevated gonadotropins due to a loss of negative feedback. Uremic patients, however, display a paradoxical mixture. Men exhibit low testosterone (a sign of peripheral failure) alongside a centrally-mediated blunted LH pulse amplitude, indicating that the pituitary response is inadequate for the degree of hypogonadism [15]. Women demonstrate a clear central failure (the absent LH surge) that prevents the function of a potentially capable ovary [30]. This dual-hit mechanism (Figure 1) explains why therapies that target only one component of the axis, such as simple testosterone replacement in men, are often insufficient [37–39]. It highlights the necessity of interventions, like intensive dialysis or transplantation, that can ameliorate the entire uremic state and thereby relieve the pathological pressure on both the central and peripheral components of the reproductive system.

BOX: key practice points: pathophysiology

- Reproductive dysfunction in dialysis is not a localized issue but a systemic endocrine disease driven by uremia.
- The ‘dual-hit’ model explains why treatments must address both central (brain) and peripheral (gonadal) dysfunction.
- Chronic inflammation and oxidative stress are key drivers of both hormonal imbalance and the vascular complications (like ED) seen in these patients.

## The male patient: a profile of uremic hypogonadism

In men with ESRD on hemodialysis, reproductive dysfunction manifests as a distinct clinical syndrome known as uremic hypogonadism. This condition is characterized by a specific pattern of hormonal derangements that drive the clinical sequelae of infertility, erectile dysfunction, and loss of libido.

### Hormonal derangements: the hypergonadotropic hypogonadal state

The hormonal milieu of the uremic male is classically described as hypergonadotropic hypogonadism, although this term does not fully convey the complexity of the underlying central defects.

Testosterone Deficiency: A deficiency in testosterone is a cardinal and highly prevalent feature, affecting an estimated 40–60% of male patients on maintenance hemodialysis. Both total and biologically active free testosterone levels are consistently found to be low or in the low-normal range. This deficiency arises from a dual insult: impaired production by testicular Leydig cells, which become resistant to LH stimulation, and potentially an increased metabolic clearance rate of the hormone [2,40,41].

Elevated Gonadotropins (LH & FSH): In response to low testosterone, the pituitary gland increases its secretion of gonadotropins, leading to elevated serum levels of LH and, to a lesser extent, FSH. This elevation, however, is not a simple compensatory mechanism. It is amplified by the fact that the diseased kidneys can no longer effectively clear these hormones from circulation, leading to a prolonged half-life. Critically, this quantitative increase in LH is accompanied by a qualitative defect: the normal high-amplitude pulsatile secretion of LH is lost, replaced by a high-frequency, low-amplitude pattern that provides a less effective stimulus to the testes. Serum FSH levels are also often normal or elevated, a finding attributed to damage to the Sertoli cells of the testes and a subsequent reduction in the secretion of inhibin, a hormone that normally provides negative feedback to the pituitary for FSH release [42–44].

Hyperprolactinemia: Elevated levels of prolactin are found in the large majority of male hemodialysis patients and represent a significant contributor to the hypogonadal state [45,46]. Prolactin is considered a functional uremic toxin in this context [47]. Its accumulation, due to both increased secretion and decreased clearance, exerts a powerful inhibitory effect on the HPG axis by suppressing GnRH release from the hypothalamus. This further dampens LH pulsatility, reduces testosterone production, and has a direct negative impact on libido and erectile function [15].

### Clinical sequelae: from cellular damage to functional impairment

The profound hormonal disturbances of uremic hypogonadism translate directly into severe clinical consequences that affect fertility, sexual function, and overall well-being.

- Impaired Spermatogenesis: The combination of a dysfunctional hormonal axis and the direct toxic effects of the uremic environment leads to significant and often irreversible damage to the testes. Histological examination reveals a spectrum of abnormalities, from a general reduction in sperm production (hypospermatogenesis) to a complete absence of sperm (azoospermia) [48]. The damage is particularly evident in the later stages of sperm maturation, which are highly dependent on adequate testosterone and FSH signaling. This results in a high rate of infertility among male dialysis patients, often accompanied by a measurable reduction in testicular volume [49,50].
- ED: ED is the most prevalent and distressing symptom of sexual dysfunction in this population. Its pathophysiology is a paradigmatic example of a multifactorial disorder, representing a convergence of the systemic complications of ESRD.
- Endocrinologic Factors: The foundational hormonal issues of low testosterone and high prolactin are major drivers, primarily by reducing sexual desire (libido) and directly interfering with the neurochemical pathways that initiate and sustain an erection [51,52].
- Vascular Factors: Uremia is a state of accelerated atherosclerosis and profound endothelial dysfunction [53]. The chronic inflammation and oxidative stress characteristic of ESRD damage the delicate lining of blood vessels throughout the body, including the penile arteries. This leads to impaired vasodilation and reduced blood inflow (arteriogenic ED) as well as failure to trap blood within the corpora cavernosa (veno-occlusive dysfunction), making it difficult to achieve and maintain a rigid erection [54]. The extremely high prevalence of vasculogenic ED, where impaired blood flow is the primary cause, should be interpreted as more than just a localized complication affecting quality of life. Erection is a sensitive hemodynamic event, and the small penile arteries can be among the first vessels in the body to manifest the clinical consequences of systemic vascular disease. Therefore, the development or worsening of ED in a male hemodialysis patient can serve as a crucial clinical marker—a ‘vascular sentinel event’—for progressing systemic endothelial dysfunction and advanced atherosclerosis. Its presence signals a heightened risk for major adverse cardiovascular events, such as myocardial infarction and stroke, and should prompt clinicians to conduct a more aggressive evaluation and management of the patient’s overall cardiovascular risk profile. This reframes ED from a lifestyle issue to a potentially vital prognostic indicator.
- Neurologic Factors: Many patients with ESRD develop uremic neuropathy, which affects both the autonomic and peripheral nervous systems [55]. Damage to the parasympathetic nerves that control penile blood flow disrupts the critical signaling required for the erectile response [56].
- Psychological Factors: The immense physical and emotional burden of living with a chronic, life-sustaining illness like ESRD leads to high rates of depression, anxiety, and poor body image, all of which are powerful independent contributors to erectile dysfunction [57,58].

BOX: key practice points: the male patient

- Screen all male hemodialysis patients for symptoms of hypogonadism and ED.
- Initial lab workup should include total testosterone and prolactin.
- Recognize that ED is multifactorial; addressing hormonal issues alone may be insufficient if significant vascular or neurologic damage is present.
- Consider ED a ‘vascular sentinel event’ that should trigger a broader cardiovascular risk assessment.

## The female patient: anovulation, amenorrhea, and infertility

For women on hemodialysis, reproductive dysfunction is dominated by a profound disruption of menstrual cyclicity, leading to a state of chronic anovulation and, consequently, a very high rate of infertility. While the clinical picture differs from that in men, the root cause is the same: the toxic and inflammatory uremic milieu causing a catastrophic failure of the HPG axis.

### Hormonal derangements: a state of anovulatory chaos

The hormonal profile of a premenopausal woman on hemodialysis is one of acyclicity and central suppression, effectively mimicking a menopausal or persistent early follicular state.

- Loss of Cyclicity and Absent LH Surge: The defining feature of female reproductive dysfunction in uremia is the complete loss of the normal, rhythmic hormonal fluctuations of the menstrual cycle [59]. The disorganized and suppressed secretion of GnRH from the hypothalamus leads to an acyclic pattern of gonadotropin release from the pituitary. This breaks the delicate positive feedback loop essential for ovulation. In a healthy cycle, rising levels of estradiol from a maturing ovarian follicle signal the pituitary to release a massive surge of LH, which triggers the follicle to rupture and release an egg. In uremic women, this LH surge is absent [7,30]. Studies have confirmed this is a central defect, as administering exogenous estrogen to these women fails to provoke the expected LH surge, demonstrating a failure at the hypothalamic-pituitary level [60].
- Aberrant Hormone Levels: The loss of cyclicity results in a static and abnormal hormonal profile. Baseline serum LH levels are chronically elevated, a result of both altered feedback mechanisms and significantly reduced renal clearance of the hormone. FSH levels are typically in the normal or slightly elevated range. Plasma estradiol levels, without the stimulus for follicular development, remain in a low-to-normal, non-fluctuating range. Consequently, progesterone levels remain consistently low, providing biochemical confirmation of persistent anovulation and the absence of a luteal phase [61,62].
- Pervasive Hyperprolactinemia: As with their male counterparts, women on hemodialysis experience a very high prevalence of hyperprolactinemia, with some reports as high as 80%. This is a major pathogenic factor, as elevated prolactin levels exert a strong suppressive effect on the HPG axis, further inhibiting the already compromised pulsatile GnRH secretion and contributing directly to the anovulatory state [63].

### Clinical sequelae: from amenorrhea to infertility

The clinical consequences of this hormonal chaos are profound, impacting menstruation, fertility, and sexual health.

Menstrual Irregularities and Amenorrhea: The most common clinical presentation is a disturbance in the menstrual pattern. While this can range from infrequent periods (oligomenorrhea) to frequent bleeding (polymenorrhea), which is reported in up to 19–47% [59] of women on maintenance hemodialysis. This condition is often referred to as a ‘reversible menopause-like state’, signifying that the ovaries have ceased their cyclical function due to the systemic uremic state, often years or even decades before the natural age of menopause.

Anovulation and Infertility: Anovulation is the direct and inevitable consequence of the absent LH surge. Without the release of an egg, conception is impossible. As a result, infertility is nearly universal among women on conventional hemodialysis, with prevalence estimates up to 92% [6]. While not an absolute barrier, spontaneous pregnancy in this population is an exceptionally rare event.

Other Aspects of Sexual Dysfunction: Beyond infertility, the uremic state and its associated hormonal changes negatively impact sexual function. The chronically low estrogen levels can lead to atrophic changes in the vaginal tissue, resulting in vaginal dryness and dyspareunia. Combined with the systemic effects of uremia, such as fatigue and depression, these factors contribute significantly to decreased libido, impaired arousal, and overall sexual dissatisfaction, further diminishing the patient’s quality of life.

A remarkable aspect of the pathophysiology in women is the apparent paradox of preserved ovarian potential. Unlike the male testis, which often sustains significant and potentially permanent histological damage from uremia, the female ovary appears to maintain its fundamental ability to function if given the proper signals. Studies have shown that when uremic women are treated with clomiphene citrate, a medication that stimulates the pituitary to release gonadotropins, their ovaries respond by producing estrogen [64]. This indicates that the primary lesion is not an intrinsic failure of the ovary itself, but rather an overwhelming central inhibition at the level of the hypothalamus and pituitary. This suggests that while the peripheral ovarian capacity is preserved, the dysfunction stems from the absence of the requisite central hormonal stimulus for ovulation. This provides a compelling explanation for why fertility can be so dramatically and rapidly restored following interventions that effectively clear the uremic milieu. Both NHD and, most notably, kidney transplantation work by ‘un-braking’ this central control system, allowing the preserved peripheral ovarian machinery to resume its normal, cyclical fun

BOX: key practice points: the female patient

- Screen all premenopausal women on hemodialysis for menstrual irregularities and symptoms of sexual dysfunction and hypoestrogenism (e.g., vaginal dryness).
- Initial lab workup should include LH, FSH, estradiol, and prolactin to confirm the anovulatory state.
- Counsel patients that infertility is primarily due to a reversible central endocrine failure, not permanent ovarian damage.
- For women desiring fertility, discuss the significant benefits of intensive dialysis and kidney transplantation early.

ction.

## Key modulators of the uremic HPG axis

While the general uremic state provides the backdrop for reproductive dysfunction, specific hormonal modulators play a disproportionately large role in orchestrating the HPG axis collapse. Hyperprolactinemia is a well-established and clinically significant factor (as mentioned above), while the role of the neuropeptide kisspeptin is an emerging area of intense research interest.

In recent years, the neuropeptide kisspeptin, and its receptor GPR54, have been identified as the master upstream regulators of the HPG axis. Kisspeptin-releasing neurons in the hypothalamus act as the critical gatekeepers of reproduction, integrating metabolic and hormonal signals to drive the pulsatile secretion of GnRH. Kisspeptin is an indispensable component for the initiation of puberty and the maintenance of normal reproductive function in both sexes [65–68].

The literature available for this review contains limited direct studies on kisspeptin levels in uremic patients. However, the pathophysiology of uremic hypogonadism, which is defined by a profound central suppression of GnRH pulsatility, strongly implicates the kisspeptin signaling system as a likely point of failure. The neurotoxic and inflammatory milieu of uremia is a prime candidate for disrupting the function of these sensitive hypothalamic neurons. Studies in related conditions of hypogonadism, such as that associated with metabolic syndrome, show altered kisspeptin levels, suggesting its involvement in metabolically-driven reproductive dysfunction [68]. In some forms of idiopathic hypogonadotropic hypogonadism, impaired GnRH neuronal function demonstrates that this pathway is a critical point of failure. Whether uremia induces a similar state of kisspeptin resistance or signaling failure is a plausible hypothesis that requires investigation [69].

The role of kisspeptin in uremic hypogonadism represents a major knowledge gap and one of the most exciting frontiers for future research in this field. Elucidating how uremic toxins and inflammation impact the kisspeptin-GnRH neuronal network could fundamentally change our understanding of the disorder. Furthermore, it could unlock entirely novel therapeutic avenues. If uremia is found to suppress endogenous kisspeptin production or create a state of kisspeptin resistance, then treatment with potent, stable kisspeptin analogues could potentially bypass this block and directly re-activate the HPG axis, offering a more physiological approach to restoring reproductive function than downstream hormone replacement.

## Systemic contributors and clinical confounders

The primary endocrine disruption of the HPG axis is amplified by systemic factors common in hemodialysis patients.

Universal in ESRD, anemia causes profound fatigue that directly impairs libido and sexual function. While correcting anemia with erythropoiesis-stimulating agents (ESAs) can improve sexual function by alleviating fatigue, the impact on sex hormone profiles is inconsistent. A significant clinical challenge exists for menstruating women, where menstrual blood loss can lead to iron deficiency and resistance to ESA therapy, creating a vicious cycle that complicates anemia management [70,71].

Deficiencies in micronutrients essential for reproductive health are common due to dietary restrictions and dialysis losses. Zinc is a vital cofactor for testosterone production and spermatogenesis. Its deficiency is directly linked to uremic hypogonadism, and supplementation has been shown in some trials to increase testosterone levels and improve sexual function [72–75]. Carnitine is depleted during hemodialysis and is crucial for cellular energy. Its deficiency contributes to muscle weakness, fatigue, and ESA-resistant anemia, all of which indirectly impair sexual function [76,77].

Comorbidities and Polypharmacy: The high prevalence of comorbidities like diabetes and hypertension independently worsens sexual dysfunction through direct vascular and neurologic damage, compounding the effects of uremia [78]. Furthermore, the extensive use of medications (polypharmacy) is a major confounder, as many common drugs, including certain antihypertensives and antidepressants, can suppress libido and erectile function [79,80]

BOX: key practice points: systemic contributors

- Optimize anemia and iron status, as fatigue is a major contributor to low libido.
- Screen for and replete nutritional deficiencies, particularly zinc in male patients with hypogonadism.
- Conduct a thorough medication review to identify and potentially substitute drugs known to cause sexual side effects..

## Patient-centered outcomes and barriers to care

Beyond the biological framework, reproductive dysfunction carries a significant psychological burden that profoundly affects quality of life [81]. Patients often experience depression, anxiety, low self-esteem, negative body image, and marital tension as a direct consequence of their sexual health issues. These psychological factors can, in turn, create a vicious cycle by further suppressing libido and erectile function.

**Figure 2.**
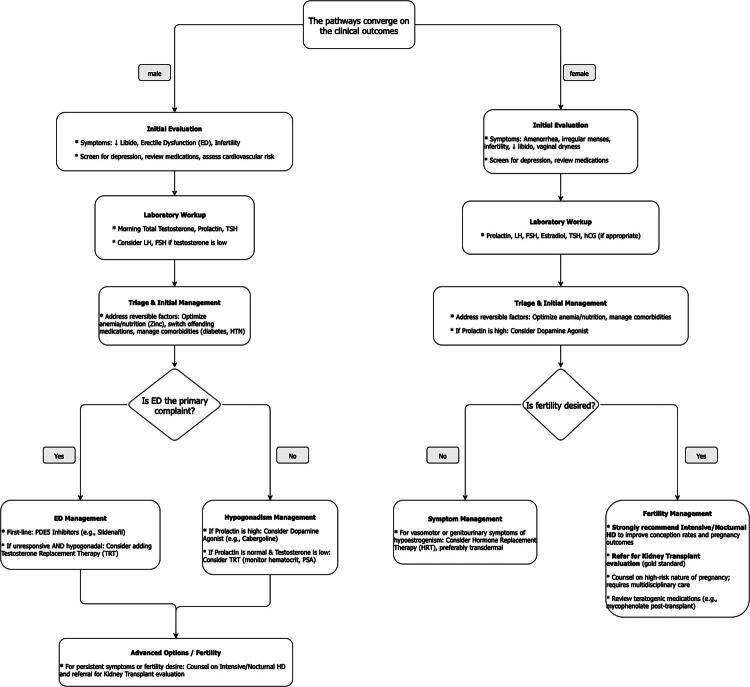
Clinical algorithm for the management of reproductive dysfunction in hemodialysis. This flowchart provides a stepwise clinical pathway for the evaluation and management of reproductive dysfunction. The algorithm begins with routine screening for all patients and then branches into separate pathways for male and female patients. Evaluation is guided by symptoms and a targeted laboratory workup. Management strategies include addressing reversible factors, initiating specific pharmacological therapies based on the underlying hormonal imbalance, and counseling on advanced therapies like NHD or kidney transplantation for patients desiring fertility. Abbreviations: ED, erectile dysfunction; TSH, thyroid-stimulating hormone; TRT, testosterone replacement therapy; PDE5i, phosphodiesterase-5 inhibitor; HRT, hormone replacement therapy; FSH, follicle-stimulating hormone; LH, luteinizing hormone.

Despite the high prevalence and impact of these issues, discussing sexual health remains a major challenge in the clinical setting. Both patients and healthcare providers face significant barriers. Patients may feel silenced by cultural norms, religious beliefs, stigma, or embarrassment. They may be unsure how to raise the topic or fear being dismissed. Clinicians, including nurses and nephrologists, often feel uncomfortable or hesitant to initiate these conversations due to a perceived lack of time, privacy, or adequate training. This mutual reluctance leads to under-diagnosis and under-treatment, leaving patients to suffer in silence. Overcoming these barriers requires a proactive, patient-centered approach that normalizes the conversation about sexual health as an integral component of comprehensive dialysis care (Figure 2).

## Therapeutic strategies: from management to restoration

The management of reproductive dysfunction in hemodialysis patients requires a multi-pronged approach that extends beyond simply addressing symptoms. The therapeutic goal should be to target the underlying pathophysiology where possible, ranging from optimizing the clearance of uremic toxins to correcting specific hormonal and nutritional deficits. The ultimate restoration of function, however, is most reliably achieved by kidney transplantation (Tables 2 and 3).

**Table 2.** Impact of dialysis modalities on reproductive endocrine function.

Modality & frequency	Clearance/dose (h/week)	Hormonal & fertility outcomes
Conventional hemodialysis (CHD)3×/week, 9–12 h	∼<20 h/week	Persistently high LH/prolactin; low sex hormones; amenorrhea 19–76.47%; poor sperm metrics
NHD 5–7×/week, ∼36–48 h/week	↑ Frequency & duration	↓ Prolactin; ↑ testosterone in men; resumption of menses; live births reported
Home Short-Daily HD ≥ 5×/week, 20–30 h/week	Moderate-intensive	Improved hormonal profile; limited pregnancy data but better than CHD

CHD: Conventional Hemodialysis; HD: Hemodialysis; LH: Luteinizing Hormone; NHD: Nocturnal Hemodialysis.

**Table 3.** Therapeutic interventions for reproductive dysfunction in hemodialysis.

Intervention	Target population	Specifics/dosing	Key benefits	Key risks & considerations
Intensive/nocturnal HD	Both	Longer sessions (6–8 hrs) – More frequent (3–7 nights/week) – Total >20–36 hrs/week	- Fertility: Increased fertility rates; restoration of regular menses. - Hormonal: Normalizes prolactin, improves testosterone. - Pregnancy: Higher live birth rates (up to 85%), longer gestation, higher birth weights.	- Increased risk of vascular access complications and infections. - May accelerate loss of residual renal function. - Increased patient/caregiver burden. - Potential for increased preeclampsia risk with >20 hrs/week.
Testosterone replacement (TRT)	Male	Injections, gels, patches, pellets.	- Function: Improves libido, mood, energy, muscle mass/strength, bone density, and anemia. – May improve ED, but less effective if severe vasculopathy exists.	- Monitoring Required: Erythrocytosis, fluid retention, prostate health (PSA/DRE). - Side effects: Testicular atrophy, gynecomastia, worsening sleep apnea, decreased HDL. - CV risk: Debated; requires careful patient selection and monitoring.
Estrogen replacement (HRT)	Female	Transdermal 17β-estradiol with cyclic progestin is a studied regimen.	- Function: Restores regular menses, improves libido and sexual function. - Hormonal: Decreases prolactin levels. - Systemic: Improves bone mineral density.	- Thrombosis Risk: Oral estrogen increases VTE risk; transdermal route is preferred due to lower risk. - Side effects: Headaches, dizziness, potential for vascular access thrombosis.
PDE5i	Male	Sildenafil, vardenafil, tadalafil.	- Function: Effective and safe for improving erectile function.	- Does not treat underlying low libido or hormonal issues. - Side effects: Headache, flushing, nasal congestion. - Interactions: Contraindicated with nitrates. Caution with alpha-blockers and post-dialysis due to hypotension risk.
Zinc supplementation	Male	Oral zinc sulfate (e.g., 250 mg/day, which provides approximately 100 mg of elemental zinc).	- Hormonal: Can increase serum testosterone and LH levels. - Function: May improve potency and frequency of intercourse in some patients.	- Evidence is based on small, older trials. - Can induce copper deficiency; monitoring of both zinc and copper is recommended. - Ineffective when added to dialysate.
Kidney transplantation	Both	Allogeneic kidney transplant (living or deceased donor).	- Gold Standard: Definitive correction of uremia and most reliable restoration of reproductive function. - Function: Rapidly restores fertility, ovulatory cycles, and libido in most patients. - Pregnancy: Superior pregnancy outcomes and live birth rates (∼72–80%) compared to dialysis.	- Major surgery with inherent risks. - Requires lifelong immunosuppression. - Some immunosuppressants are teratogenic or impair fertility. - Irreversible testicular damage may persist. - Pregnancy remains high-risk (preeclampsia, preterm birth, etc.).

CV: Cardiovascular; DRE: Digital Rectal Examination; HD: Hemodialysis; HDL: High-Density Lipoprotein; HRT: Hormone Replacement Therapy; PDE5i: Phosphodiesterase-5 Inhibitors; PSA: Prostate-Specific Antigen; TRT: Testosterone Replacement Therapy; VTE: Venous Thromboembolism.

### Optimizing renal replacement: the endocrine benefits of nocturnal hemodialysis

The observation that reproductive dysfunction correlates with the ‘dose’ of uremia provides a strong rationale for the use of more intensive dialysis modalities (e. g. nocturnal hemodialysis (NHD)). By providing enhanced clearance of small, middle, and protein-bound uremic toxins, intensive dialysis modalities aim to create a more physiological internal environment, thereby relieving the inhibitory pressure on the HPG axis.

NHD, which involves longer sessions (6–8 h) performed more frequently (3–7 nights per week), exemplifies this approach. Clinical evidence strongly supports its benefits for reproductive health [82,83]. Studies of NHD cohorts have demonstrated a remarkable increase in fertility rates; one center reported a conception rate of 15.6% among women of childbearing age on NHD, a figure dramatically higher than that seen with conventional HD [84]. Furthermore, NHD has been associated with the restoration of regular menstrual cycles, normalization of prolactin levels, improved testosterone levels in men, and significantly better pregnancy outcomes, including longer gestations, higher birth weights, and fewer maternal and fetal complications. These findings suggest that intensifying the dialysis prescription is a powerful therapeutic tool for improving endocrine and reproductive function [85–87].

### Pharmacological interventions: a critical review

Hormone Replacement Therapy (HRT) in Men: The use of testosterone replacement therapy (TRT) in hypogonadal male patients can be effective in improving libido, mood, muscle mass, and anemia. However, it must be prescribed with caution [88,89]. Potential adverse effects include erythrocytosis, fluid retention, and concerns about stimulating the growth of subclinical prostate cancer [90,91]. Moreover, TRT may not fully resolve ED if there is significant underlying vascular or neurologic damage, as it primarily addresses the desire and hormonal components of sexual function.

HRT in Women: For pre-menopausal women with amenorrhea and documented estrogen deficiency, HRT can be highly beneficial. A study using transdermal 17β-estradiol demonstrated that HRT successfully restored regular menses, led to a marked improvement in libido and sexual activity, significantly decreased serum prolactin levels, and had a positive effect on bone mineral density, helping to prevent premature osteoporosis [92]. Non-oral routes of estrogen administration (e.g., transdermal patches) are generally preferred to minimize the risk of venous thromboembolism, which is already elevated in this patient population [30].

Phosphodiesterase-5 Inhibitors (PDE5i): Medications such as sildenafil and vardenafil are established first-line treatments for ED in the general population and have been shown to be effective and safe in male hemodialysis patients. They work by enhancing the vasodilatory effects of nitric oxide in the penis, directly improving erectile mechanics. However, they do not address the underlying issues of low libido, hormonal imbalance, or systemic vascular disease [93,94].

Dopamine Agonists: For patients with significant hyperprolactinemia, dopamine agonists like bromocriptine or cabergoline can be used to lower prolactin levels. By reducing the inhibitory effect of prolactin on the hypothalamus, these agents can help to improve the function of the HPG axis and have been shown to be effective in dialysis patients [95,96].

### Nutritional supplementation: targeting reversible deficiencies

As discussed, zinc deficiency is a common and reversible contributor to hypogonadism in male hemodialysis patients. Multiple studies have investigated the utility of oral zinc supplementation. While the evidence base consists of smaller trials, the results are promising. Oral zinc administration (e.g., 250 mg/day of zinc sulfate, which provides approximately 100 mg of elemental zinc) has been shown to significantly increase serum zinc levels, which in turn leads to significant increases in plasma testosterone and LH levels [74,75]. Clinically, this has translated into improvements in potency and the frequency of sexual intercourse. Given its low cost and favorable safety profile, zinc supplementation represents a rational and potentially valuable adjunctive therapy for male patients with documented deficiency and sexual dysfunction.

### Kidney transplantation: the gold standard for endocrine and reproductive restoration

Kidney transplantation remains the definitive treatment for ESRD and offers the most profound and reliable restoration of reproductive function. By replacing the function of the failed kidneys, a successful transplant corrects the uremic milieu, clears retained toxins, resolves chronic inflammation, and allows the body’s endocrine systems to normalize [97,98].

The impact on the HPG axis is often rapid and dramatic. In many patients, fertility is restored within 6 to 8 weeks of transplantation. In men, serum testosterone, LH, and prolactin levels typically normalize, and spermatogenesis can show significant improvement, although some degree of irreversible testicular fibrosis may persist in men who were on dialysis for a long time. In women, the return of normal hypothalamic-pituitary signaling leads to the resumption of regular, ovulatory menstrual cycles and a restoration of fertility. Consequently, pregnancy outcomes are vastly superior for transplant recipients compared to patients on dialysis, with live birth rates that can approach those of the general population [99,100].

Despite these remarkable benefits, transplantation is not a panacea. The health of the allograft, the ongoing need for immunosuppressive medications (some of which, like mycophenolate, are teratogenic and must be stopped well before conception), and the presence of preexisting comorbidities continue to make pregnancy a high-risk endeavor that requires careful planning and multidisciplinary management [101,102].

BOX: key clinical pearls

- Erectile dysfunction in male hemodialysis patients is a sentinel marker for systemic vascular disease and warrants cardiovascular risk assessment.
- Hyperprolactinemia is a common, potent, and drug-treatable cause of HPG axis suppression; check prolactin levels before labeling dysfunction as refractory.
- Female infertility in hemodialysis is primarily due to central hypothalamic-pituitary inhibition, not irreversible ovarian failure, and is often reversible with kidney transplantation.
- Intensifying dialysis (e.g., nocturnal hemodialysis) is an underutilized strategy that can partially restore fertility and significantly improve pregnancy outcomes.
- For men with hypogonadism and erectile dysfunction unresponsive to PDE5i alone, combination therapy with testosterone replacement can improve efficacy.
- Always perform a medication review, as common drugs (e.g., certain antihypertensives, antidepressants) can cause or exacerbate sexual dysfunction.
- Open, patient-centered communication is essential to overcome psychological and cultural barriers to discussing sexual health.

## Conclusion: clinical practice summary

Reproductive dysfunction is a pervasive and multifactorial complication of hemodialysis, driven primarily by a ‘dual-hit’ endocrine collapse of the HPG axis. For clinicians, this mandates a proactive and integrated approach. Routine screening for sexual and reproductive health issues should be standard practice for all dialysis patients, as these concerns are often unspoken but have a major impact on quality of life. The initial evaluation should be guided by the patient’s sex and symptoms, typically involving a hormonal panel (testosterone, prolactin, gonadotropins) and a thorough review of comorbidities and medications.

For male patients, it is crucial to recognize that ED serves not only as a quality-of-life concern but also as a potent prognostic marker, acting as a ‘vascular sentinel event’ that warrants a comprehensive cardiovascular risk assessment.

Management should be stepwise and patient-centered. Addressing reversible factors like anemia, nutritional deficiencies (e.g., zinc), and offending medications is a crucial first step. For specific dysfunctions, targeted therapies such as PDE5i for ED, dopamine agonists for hyperprolactinemia, and hormone replacement for symptomatic hypogonadism are effective. Critically, clinicians should recognize the limitations of conventional HD and counsel eligible patients on the profound benefits of more intensive dialysis modalities and kidney transplantation, which remain the most effective strategies for restoring fertility and normalizing endocrine function. A multidisciplinary approach that addresses both the physiological and psychological dimensions of this condition is essential for providing holistic care.

## Future directions: a research agenda

Despite significant advances, critical knowledge gaps remain. A clear research agenda is needed to move the field forward.

Temper Speculation on Kisspeptin: The role of the kisspeptin system in uremic hypogonadism is a promising but speculative frontier. A structured research agenda is required to test this hypothesis. This should include prospective studies to measure kisspeptin levels in incident dialysis patients and pilot randomized controlled trials to assess the response to kisspeptin analogues. Such studies would be essential to confirm or falsify its role and therapeutic potential.

Emphasize Emerging Therapies:

Kisspeptin Analogues: With several agonists and antagonists in clinical trials for other reproductive disorders, these agents represent a novel therapeutic class that could potentially restore central HPG axis signaling in uremic patients.

Continuous Clearance with Wearable/Implantable Artificial Kidneys: The development of next-generation technologies, such as wearable or implantable artificial kidneys, aims to provide more continuous and physiological clearance of uremic toxins. By more closely mimicking native kidney function, these devices could prevent or fully reverse the endocrine derangements of uremia, though they are still in early phases of development and not yet FDA-approved.

AI-Driven Reproductive Health Monitoring: The large datasets generated during dialysis are ideal for artificial intelligence (AI) applications. AI-driven algorithms could help predict hormonal fluctuations, optimize anemia management, and personalize treatment plans to improve reproductive health outcomes, though this field is still in its infancy.

## Data Availability

No datasets were generated or analyzed during the current study.
